# Impulsive Buying and Deferment of Gratification Among Adults With ADHD

**DOI:** 10.32872/cpe.9339

**Published:** 2024-09-30

**Authors:** Sverrir Björn Einarsson, Baldur Heiðar Sigurðsson, Sigurlín Hrund Kjartansdóttir, Páll Magnússon, Jón Friðrik Sigurðsson

**Affiliations:** 1 Department of Psychology, Reykjavík University, Reykjavík, Iceland; 2 Landspítali – The National University Hospital of Iceland, Reykjavík, Iceland; 3 The Health Institute of East Iceland, Egilsstaðir, Iceland; 4 Faculty of Medicine, University of Iceland, Reykjavík, Iceland; Friedrich-Alexander-Universität Erlangen-Nürnberg, Erlangen, Germany

**Keywords:** ADHD, impulsivity, impulsive buying, deferment of gratification, mediation analysis

## Abstract

**Background:**

Impulsivity symptoms have been studied thoroughly in adults with ADHD, including hasty actions and decisions without considering possible consequences. The objective of our study was to investigate impulsive buying and deferment of gratification among adults with ADHD and a comparison group.

**Method:**

The participants were 225 adults with ADHD and 121 university students who completed the Buying Impulsiveness Scale (BIS), the Deferment of Gratification Questionnaire (DOGQ), the Adult ADHD Rating Scale—IV (ADHD-RS), as well as background questions.

**Results:**

Significant differences were found between the two groups on the three scales, the ADHD group showing more ADHD symptoms, more frequent impulsive buying behaviour and less ability to defer gratification. Mediation analyses yielded significant indirect effects in both samples, which suggests that the relationship between ADHD symptoms and impulsive buying is mediated by the ability to defer gratification.

**Conclusion:**

The results suggest that placing emphasis on improving the capacity of adults with ADHD to defer gratification might be beneficial in treatment.

## Highlights

- Adults with ADHD symptoms are more likely to show impulsive buying behavior and lesser ability to defer gratification than those without ADHD symptoms.
- The relationship between ADHD symptoms and impulsive buying is mediated by the ability to defer gratification.
- Improving capacity to defer gratification should be considered in treatment of adults with ADHD.
- More research on impulsive buying in ADHD is warranted. Future research might examine online impulsive buying among adults with ADHD.

ADHD (attention deficit hyperactivity disorder) is a developmental disorder characterized by inattention, hyperactivity and impulsivity, that is inconsistent with the development and age of the adult (American Psychiatric Association [APA], 2013). The prevalence rate of ADHD is around 5% amongst children and 2.5% in adults when diagnoses are made according to the *Diagnostic and Statistical Manual of Mental Disorders, Fifth Edition* (DSM-5) (APA, 2013). Several twin studies indicate a strong genetic component in 70-95% of cases (Comings, 2001).

Impulsivity, a core symptom of ADHD, has been conceptualized as: (a) decreased sensitivity to negative outcomes of behaviour, (b) rapid, unplanned, reactions to stimuli before complete processing of information, and (c), lack of regard for long-term consequences (Moeller et al., 2001). Impulsivity symptoms have been extensively studied in adults with ADHD and among them are hasty actions and decisions without considering possible effects or consequences (APA, 2013; Barkley, 1997). Symptoms of impulsivity and hyperactivity are expected to decrease during adolescence and early adulthood (Biederman et al., 2000), but some studies have shown that impulsivity persists into adulthood and may be a core factor of many behavioural dysfunctions seen in adults with ADHD (Asherson et al., 2016; Babinski et al., 1999).

Impulsive buying has been conceptualized as “a sudden, often powerful and persistent urge to buy something immediately” (Rook, 1987), and studies indicate that impulsive buying tendencies derive from problems with executive functions such as problem solving, planning skills, reactivity, inattention and inflexibility (Arican & Kafadar, 2022). It has also been suggested that motor and non-planning impulsivity or lack of self-control are important aspects of impulsive buying (Baumeister, 2002; Sokić & Korkut, 2020). Deferment of gratification is a component of self-control and is based upon resisting the urge to receive an immediate reward in the hope of receiving a more valuable reward in the future (Mischel et al., 1989). The ability to defer gratification is fundamental to effective self-control as studies suggest that consumers with greater self-regulatory resources are more likely to resist impulsive buying and there are indications that impulsive buying is motivated by immediate gratification (Badgaiyan et al., 2016; Roberts & Manolis, 2012; Sun et al., 2004; Vohs & Faber, 2007).

As of yet, no studies have demonstrated the relationship between ADHD symptoms and impulsive buying among those with ADHD, although a few studies indicate that ADHD symptoms affect personal finances. Bangma et al. (2019) explored problems in multiple domains of everyday life, including financial decision-making, among adults diagnosed with ADHD. The results show that compared with healthy controls, those diagnosed with ADHD reported a poorer financial situation, more debt, a lower incidence of having a savings account and a greater tendency to buy on impulse. A recent study by Koerts et al. (2021) explored financial judgment among adults with ADHD. They found that adults with ADHD had lower financial competence scores than those without ADHD on appreciation, reasoning, understanding and communication. In addition, Barkley et al. (2006) found that compared with controls, adults with ADHD had more difficulties in allocating funds, i.e., problems with saving money, paying bills on time and a greater tendency for recklessness and impulsive buying. The same study found that growing up with ADHD is a risk factor for financial problems in adulthood, regardless of whether ADHD symptoms persist into adulthood although the risk was even greater in those cases where the symptoms did persist into adulthood.

Adults who are highly impulsive buyers tend to be more emotionally attracted to the item they are buying and more likely to desire immediate gratification (Hoch & Loewenstein, 1991). Jackson and MacKillop’s (2016) meta-analysis on the relationship between ADHD and defer discounting indicates that people with ADHD have a greater tendency to choose immediate and less valuable rewards instead of later rewards with more value, compared with people without the disorder.

The aim of this study was to investigate impulsive buying as a function of ADHD symptoms and the ability to delay gratification. Of particular interest are the possible mediating effects of defer of gratification on the relationship between ADHD and impulsive buying. Three predictions were made: 1) adults diagnosed with ADHD will have higher levels of impulsive buying than a sample of normal controls, 2) adults diagnosed with ADHD will have more difficulty to defer gratification than normal controls, and 3) the link between ADHD and impulsive buying is mediated by the ability to defer gratification.

## Method

### Participants

The participants consisted of two groups: (1) a sample of 226 adults diagnosed with ADHD and (2) a comparison sample (non-ADHD group) of 134 university students at Reykjavik University. Inclusion criteria for both groups were: (a) age between 18 and 65 years, (b) reporting if and where the ADHD diagnosis was made. In the student sample, 12 participants responded with “yes” to the question about ADHD diagnosis and were therefore eliminated from the sample, leaving a student sample of 122. After screening for outliers and influential cases (see below) two statistical outliers were identified and eliminated from the dataset, one from each sample, leaving a student sample of 121 participants and a clinical sample of 225 for the final analysis.

The ADHD sample had a mean age of 35.72 (*SD* = 9.80) and consisted of 162 (72.00%) females (mean age 35.09, *SD* = 9.08) and 63 (28.00%) males (mean age 37.33, *SD* = 11.37). One-hundred-fifty-five (68.89%) claimed to have graduated from upper secondary school and 187 (83.11%) being employed or studying at the time of the study. A majority (81.78%) of the ADHD group reported having received their diagnoses from psychologists or psychiatrists in private practice, 10.22% from the ADHD team at Landspítali – The National University Hospital of Iceland, 4.00% from institutes of child mental health and developmental surveillance, and 4.00% from educational psychologists. It is worth mentioning that the diagnostic process of ADHD in private practice in Iceland is common, and that psychiatrists and psychologists are expected to follow clinical guidelines issued by the Directorate of Health (Baldursson et al., 2012).

The non-ADHD sample had a mean age of 24.57 (*SD* = 5.33) and consisted of 82 (68.33%) females (mean age 25.00, *SD* = 6.07) and 38 (31.67%) males (mean age 23.54, *SD* = 2.96). One participant in the comparison group did not specify gender. The majority (119; 98.35%) claimed they had graduated from upper secondary school and all of them were university students at the time of the study.

### Measures

#### The Buying Impulsiveness Scale (BIS)

The BIS (Rook & Fisher, 1995) was designed to measure impulsive buying behaviour and contains nine statements such as, “I often buy things without thinking,” and “I carefully plan most of my purchases.” The participant indicates how much he or she agrees with these statements on a 5-point Likert scale (1 = strongly disagree to 5 = strongly agree). The score of one item was reversed. Higher scores indicate more impulsive buying tendencies. The scale has shown high levels of reliability (α = .88) (Rook & Fisher, 1995). It was translated to Icelandic especially for this study using accepted methods, i.e. three independent forward translations which then were compared and semantic differences resolved arriving at the single translation which then was back-translated and amended accordingly (Gudmundsson, 2009).

#### The Deferment of Gratification Questionnaire (DOGQ)

The DOGQ (Ray & Najman, 1986) was designed to measure deferment of gratification in relation to financial planning and contains 12 questions such as “Are you good at saving your money because you have had to wait for it and plan for it?” and “Do you like to spend your money as soon as you get it?” The participant indicates how much he or she agrees with these statements on a 7-point Likert scale (1 = Very strongly disagree, 4 = Sometimes, 7 = Very strongly agree). The scores of six items were reversed. Lower scores indicate more difficulty in deferring gratification. The original scale has acceptable internal consistency (α = .72) (Ray & Najman, 1986). The scale was translated specifically for this study using three independent forward translations which were compared and semantic differences resolved arriving at the single translation which then was back-translated and amended accordingly (Gudmundsson, 2009).

#### The Adult ADHD Rating Scale—IV (ADHD-RS)

The ADHD-RS (Magnússon et al., 2006), was designed to measure the symptoms of attention-deficit/hyperactivity disorder (ADHD). It contains 18 statements, nine items of inattention, five of hyperactivity, and four of impulsivity symptoms. The frequency and severity of each item is rated for the past six months on a 4-point Likert scale (0 = Never or rarely, 1 = Sometimes, 2 = Often, 3 = Very often). A higher score indicates more ADHD symptoms. The total score consists of 18 items, the scores of two subscales, the inattentive subscale (ADHD-I) and the hyperactivity/impulsivity subscale (ADHD-H/I). The scale has shown good reliability and validity and strong correlation with informal ratings of symptoms and interview-based diagnoses in childhood and adulthood. The scale was translated into Icelandic by one of the authors of this paper and its validity and reliability turned out to be satisfactory (Magnússon et al., 2006).

#### The Background Information Questionnaire

The questionnaire developed by the ADHD Clinic at Landspítali – The National University Hospital of Iceland, consists of questions about gender, age, education, employment and ADHD diagnosis.

### Procedure

A link to a survey (SurveyMonkey), was emailed by the Icelandic ADHD Organisation, association of the ADHD community in Iceland, to all of its members and posted on its official Facebook page (https://www.facebook.com/ADHDsamtokin/?locale=is_IS). For the comparison group, the survey was administered on paper in class in Computer Science, Law, Business and Sports Science at Reykjavik University. Participation of both groups was anonymous and voluntary and filling out the survey was considered as an informed consent for both groups as they had previously received written information about the study.

The study was approved by Reykjavik University and the Icelandic Bioethics Committee (no. 18-0-51).

### Statistical Analysis

The data were analysed using the SPSS (v. 28.0). Descriptive statistics were calculated on the measures used in the main analysis and *t*-tests were carried out to see if the ADHD sample differed from the student sample on those variables. To adjust for the inflated Type I error rate associated with multiple testing, Bonferroni correction was employed resulting in an alpha level of 0.016 instead of 0.05. In the main analyses Hayes’s SPSS PROCESS Macro v. 4.2 (Hayes, 2018) Model 4 was used to carry out three identical mediation analyses: one for the total sample and one for each of the subsamples. This was done to examine whether the assumed effect of ADHD on impulsive buying was mediated by the ability to defer gratification.

The method relies on a series of regression analyses outlined by Baron and Kenny (1986). The total effect of the predictor (in this study ADHD-RS) on the outcome variable (BIS) is first assessed while leaving other variables out of the model. This is then followed by examining the same relationship while controlling for the presumed mediator (DOGQ), taking into account the relationship between the predictor and the mediator.

According to Baron and Kenny, if an originally significant total effect becomes insignificant when controlling for the mediator, mediation is said to have occurred, i.e. the predictor operates through the mediator to affect the outcome. Mediation is considered partial if some measurable, albeit insignificant relationship is left to account for, but if it reduces to zero when controlling for the mediator, the relationship between the predictor and the outcome is said to be completely mediated.

**Figure 1:**
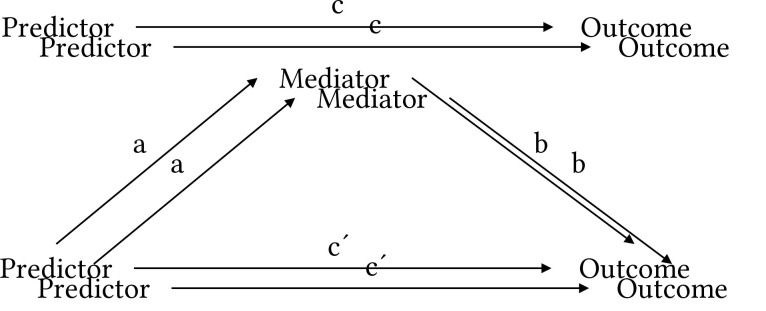
A Conceptual Model of Mediation *Note.* c represents the total effect, c´ represents the direct, unmediated portion of the total effect and a and b, through the mediator represents the indirect, mediated portion of the total effect.

The total effect (path c in Figure 1) is thus broken down into a direct effect (path c´, the effect not accounted for by the relationship between the predictor and the mediator) and an indirect effect (paths a and b through the mediator, the effect accounted for by the relationship between the predictor and the mediator, or the mediated effect). These two regression analyses were, as mediation was originally defined, considered enough to calculate the indirect effect simply by subtracting the direct effect from the total effect. This, however, assumes the predictor and the mediator do not interact to affect the outcome. To account for a possible interaction effect, the indirect effect was therefor soon defined as the product of a) the first order relationship between the predictor and the mediator and b) the relationship between the mediator and the outcome when controlling for the predictor. In case of no interaction, these two methods (c-c´, and axb) yield the same result.

Therefore, for a complete mediation analysis, three regression analyses are needed to calculate the indirect effect. First, to assess the total effect (path c), the outcome is regressed on the predictor. Second, to assess the direct effect of the predictor (path c´), the outcome is regressed on both the predictor and the presumed mediator. The direct effect is thus the regression coefficient of the predictor when controlling for the mediator. In this second regression analysis, we also get the first term needed to calculate the indirect effect, namely the effect of the mediator on the outcome when controlling for the predictor (path b). Finally, to calculate the second term needed for the indirect effect (path a), the presumed mediator is regressed on the predictor.

As mentioned above, Baron and Kenny (1986) conceptualised mediation as an originally significant effect of a predictor on an outcome becoming insignificant (or zero) when controlling for a mediating variable. More recently these requirements of (non-)significance of relationships have come under scrutiny. For instance, Zhao et al. (2010) argue that indirect effects are interpretable and meaningful regardless of the significance of the relationships between the predictor and outcome. We therefor focus on the indirect effect but present the whole model as it needs to be considered when interpreting the meaning of the indirect effect.

Various methods of testing the significance of the indirect effect have been proposed. In this study the significance was examined by using bootstrapping procedures (5,000 bootstrap samples) to apply 95% bias corrected accelerated confidence intervals (95% BCa CI) around the estimate of the indirect effect. If the 95% BCa CI contains zero, the indirect effect is insignificant, and mediation cannot be assumed. Finally, standardized indirect effects were calculated to give an impression of the sizes of the indirect effects. In all three analyses the ADHD-RS was the predictor variable, the BIS the outcome variable and the DOGQ the mediating variable.

Before any analyses were conducted the dataset was screened for outliers using Mahalanobis’s distance (critical value at alpha level 0.001), Cook’s distance (critical value = 1) and centred leverage values (critical value at 2(k+1)/n). These values were obtained by running a regression analysis in each of the samples separately with both the predictor and the mediator in the model. Participants who reached the critical values on two of these measures were considered statistical outliers and were eliminated from the sample.

## Results

### Group Differences in Symptom Severity, Ability to Defer Gratification and Impulsive Buying

Table 1 shows descriptive statistics for the measures used in the mediation analyses as well as their reliabilities. All the measures have acceptable to excellent reliabilities. The means differ significantly between the two groups in the predicted directions, the ADHD group having a lower mean on the DOGQ, *t*(334) = -18.34, *p* < .001, and higher means on the ADHD-RS, *t*(341.39) = 32.97, *p* < .001, and BIS, *t*(295.86) = 13.28, *p* < .001.

**Table 1:** Descriptive Statistics and Cronbach’s Alpha for the ADHD-RS, the DOGQ and the BIS for the ADHD and the Non-ADHD Groups

Measures	*N*	*M*	95% CI		*SD*	Cronbach’s alpha
			*LL*	*UL*		
ADHD-RS						
Non-ADHD	121	7.88	7.00	8.76	4.89	
ADHD	224	31.59	30.48	32.70	8.46	
Total	345	23.27	21.84	24.71	13.53	0.95
DOGQ						
Non-ADHD	117	58.03	56.37	59.69	9.07	
ADHD	219	38.21	36.93	39.49	9.62	
Total	336	45.11	43.68	46.54	13.35	0.84
BIS						
Non-ADHD	120	19.57	18.35	20.79	6.75	
ADHD	221	30.78	29.64	31.91	8.57	
Total	341	26.83	25.81	27.86	9.61	0.93

*Note.* CI = Confidence interval; ADHD-RS = The Adult ADHD Rating Scale; DOGQ = Deferment of Gratification Questionnaire; BIS = Buying Impulsiveness Scale.

### Mediation Analyses

**Figure 2:**
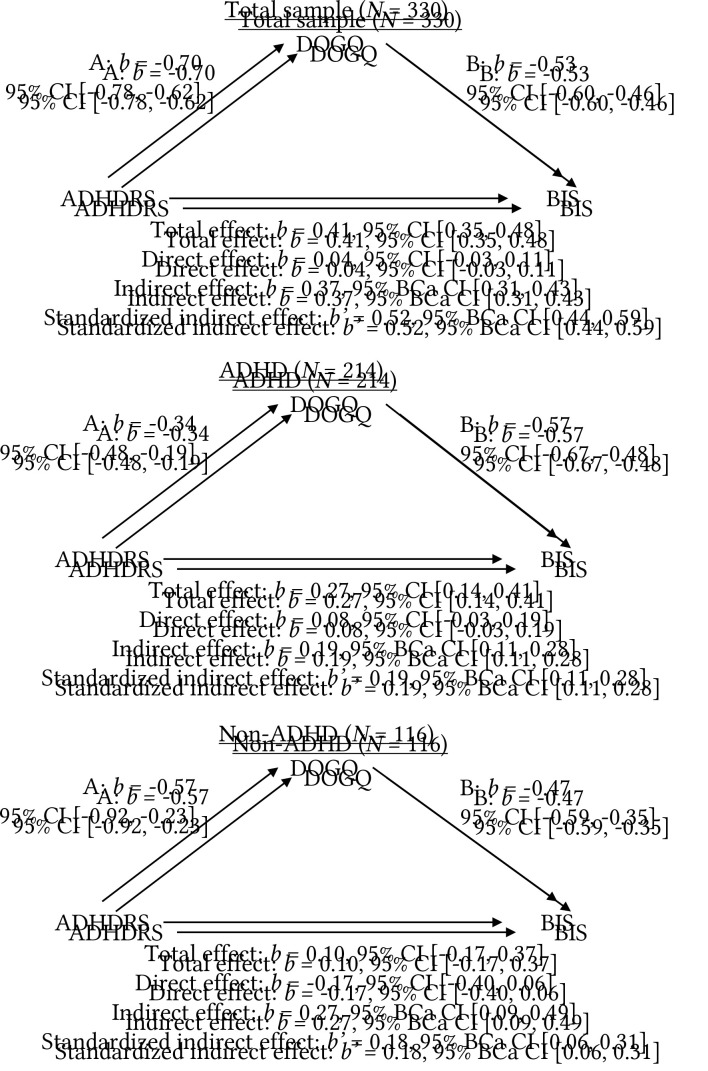
Model for the Mediation of the Association Between ADHD Symptoms and Impulsive Buying Behaviors Through the Ability to Defer Gratification *Note.* CI = Confidence interval; BCa CI = Bias Corrected and accelerated Confidence Interval; ADHD-RS = The Adult ADHD Rating Scale IV; DOGQ = Deferment of Gratification Questionnaire; BIS = Buying impulsiveness Scale.

As shown in Figure 2 the same pattern emerges in all three mediation models, where there is a substantial drop from the total effect to the direct effect, although neither effect is statistically significant in the non-ADHD sample. In the other two samples the total effect of ADHD is significant but becomes insignificant when controlled for the ability to defer gratification. All three 95% BCa CIs indicate significant indirect effects, suggesting a mediating effect of the ability to defer gratification. The fit of the models were assessed by calculating *R*^2^ when both the predictor and the mediator are included. In the total sample *R*^2^ = 0.60, in the ADHD sample 0.45 and non-ADHD sample 0.36.

## Discussion

Impulsivity symptoms in ADHD have been extensively studied (Barkley, 1997) and research has shown the disorder to be linked to various financial problems. It is therefore noteworthy that to date, there are to our knowledge no studies on the relationship between ADHD and impulsive buying, a type of impulsive behaviour that could clearly contribute to claimed financial problems. There is, however, research indicating that self-regulation and the ability to defer gratification may bolster against such impulsive buying in the general population (Badgaiyan et al., 2016; Roberts & Manolis, 2012; Sun et al., 2004). In this study we compared a group of people diagnosed with ADHD to a student sample, predicting higher levels of impulsive buying and less ability to defer gratification among the ADHD sample. Our main analysis, however, tested a mediational model where it was hypothesised that this link between ADHD and impulsive buying would be mediated by the ability to defer gratification.

As predicted, significant differences were found between the two groups on measures of ADHD symptoms, deferment of gratification and impulsive buying, with the ADHD group showing more ADHD symptoms, more frequent impulsive buying behaviour and less ability to defer gratification. Previous studies reported in Jackson and MacKillop’s (2016) meta-analysis suggest that those with ADHD have a greater tendency to choose immediate and less valuable rewards instead of later rewards, while other studies indicate that deficits in self-regulation among non-ADHD adults can increase the risk of impulsive buying (Roberts & Manolis, 2012; Vohs & Faber, 2007).

However, our main findings from the mediation analyses indicate an indirect relationship between ADHD symptoms and impulsive buying mediated by deferring gratification, suggesting that the ability to defer gratification may be an important mechanism through which ADHD exerts its effect on impulsive buying. Moreover, in the total sample and the ADHD sample, the total relationship between ADHD symptoms and impulsive buying was completely eliminated when the relationship between ADHD and the ability to defer gratification was accounted for, suggesting that no other mediators are needed to account for the effect of ADHD on impulsive buying.

In the student sample however, neither the total relationship nor the direct relationship was significant. This makes the significant indirect relationship somewhat difficult to interpret, although the results could be due to low power as the sample was substantially smaller than the ADHD sample and a larger sample might yield either a significant total effect, direct effect, or both. In that case, and assuming the same trends, the seamingly paradoxically negative direct relationship between ADHD symptoms and impulsive buying in the student sample (meaning that more symptoms is related to less impulsive buying) could possibly be understood if we assume that the impaired ability to defer gratification is a stronger force in an ADHD population than in a healthy population due to impairment in executive functions, commonly associated with ADHD. It would therefore be interesting to repeat this study with a larger normal sample to get more clarity regarding the meaning of the mediation through the ability to defer gratification.

These results add to the results of Sun et al. (2004), which reported that impulsive buying behaviour is motivated by immediate gratification. The results suggest that improved ability to defer gratification may be beneficial for people in general (just as re-evaluation of negative thoughts can benefit people in general, not only people with emotional problems) and for people with ADHD in particular, for whom impulsive buying may be a serious problem.

There are some limitations to the current study. As the participants in the ADHD sample were recruited via the internet and email, the accuracy of their ADHD diagnoses could not be clinically ascertained. Also, the groups were different in age, education level and employment, the comparison group being significantly younger on average and with smaller age variation, and the non-ADHD group reporting higher educational level and being more actively studying or working than the ADHD group. A final limitation is that although mediational models assume causality with a specified causal direction, the design of the study does not allow any causal inferences. It would therefore be of interest to conduct an experiment to see if a treatment intervention targeting the ability to defer gratification would in turn also affect overall ADHD symptoms. Such an experiment might help to establish causality.

The study has several strengths. Among them is a large sample size, increasing both reliability and generalizability of the results. The relationship between ADHD symptoms and impulsive buying, mediated by deferment of gratification, has to our knowledge not been investigated before so our findings are an important addition to the existing literature on the relationship between ADHD and impulsive buying.

Future research should examine online impulsive buying among adults with ADHD as products and services are increasingly becoming more accessible and cost-saving through online shopping, which may trigger impulsive buying among consumers (Saha et al., 2020; Sun & Wu, 2011).

## Data Availability

The data are not available for open access.

## Social Media Accounts

@Sverrirbjorn, @fri_jonfsig

## Reporting Guidelines

The Publication Manual of the American Psychological Association.

## Preregistration

The study was not pre-registered but approved by the relevant authorities and the Icelandic Bioethics Committee (no. 18-0-51).
